# Encapsulation of ultrasound-assisted flaxseed meal protein hydrolysate in nanoliposomal systems by new formulation: Physicochemical properties, release behavior under simulated gastrointestinal conditions

**DOI:** 10.1016/j.fochx.2025.102389

**Published:** 2025-03-25

**Authors:** Faezeh Farzanfar, Alireza Sadeghi Mahoonak, Mohammad Ghorbani, Seyed Hossein Hosseini Ghaboos, Shima Kaveh

**Affiliations:** a Faculty of Food Science & Technology, Gorgan University of Agricultural Sciences and Natural Resources, Gorgan 4918943464, Iran; b Food Science and Technology Research Center of East Golestan, Azadshahr Branch, Islamic Azad University, Azadshahr, Iran

**Keywords:** Flaxseed, Hydrolyzed protein, Nanoliposome, Antioxidant, Ultrasound

## Abstract

Flaxseed meal is rich in protein with antioxidant properties. In this study, its protein was hydrolyzed using alcalase and pancreatin (1.2 %–3 %) after ultrasonic pretreatment. The pancreatin-treated hydrolysate showed the highest antioxidant activity, with 75.66 % DPPH inhibition, 70.39 % iron ion chelation, and a total antioxidant activity absorption value of 0.86 nm. To enhance antioxidant stability and control release, the hydrolyzed protein was encapsulated in liposomal nanovesicles with varying flaxseed oil (0.01, 0.02 and 0.03 g/10 ml chloroform) and cholesterol (0.02, 0.03, 0.04 and 0.05 g) concentrations. Treatment L3 (0.02 g flaxseed oil) exhibited the best antioxidant and physicochemical properties, with 95.64 % encapsulation efficiency, 409 nm particle size, −15.9 mV zeta potential, 87.51 % DPPH inhibition, 56.60 % iron ion chelation, and 1.339 total antioxidant activity absorption at 695 nm. The results showed that the optimal treatment of this study (L3) can be a suitable alternative to synthetic antioxidants in food formulations.

## Highlights

• Hydrolyzed flaxseed meal protein offers an alternative to industrial antioxidants.
• Nanoliposomes improve protein stability within the digestive system.
• Flaxseed oil is a suitable substitute for cholesterol in nanoliposome formulations.

## 1 Introduction

Reactive oxygen species (ROS) or free radicals are produced during normal metabolism through oxidation reactions. Excessive amounts of ROS cause oxidation and harmful effects on human health (Zhang et al., 2024). Recently, the possible toxicity and adverse effects of synthetic antioxidants have raised concerns (Gurusaran et al., 2024).

In recent years, researchers have identified antioxidant bioactive peptides from animal and plant protein sources (Zhu et al., 2022). These peptides are short sequences of 2 to 50 amino acids that present in their precursor protein in an inactive form and exhibit various biological functions (González-Osuna et al., 2024). The release of bioactive peptides from their precursor proteins through enzymatic hydrolysis of protein is influenced by several factors, such as hydrolysis time, pH, temperature, and enzyme-to-substrate ratio (Auwal et al., 2017).

Bioactive peptides can be produced from various sources; in this regard, waste materials from the food processing industry are economically and environmentally favorable (Zhu et al., 2022). Flaxseed meal is the primary by-product of flaxseed oil extraction and is rich in protein (Hoshiar et al., 2021). Studies show that flaxseed peptides exhibit various physiological properties, such as antibacterial activity (Heymich et al., 2021), inhibition of angiotensin-converting enzyme (ACE) (Li et al., 2024), antioxidant activity (Yekta et al., 2020), and antidiabetic effects (del Carmen Hernández-Barillas et al., 2025; Xue et al., 2017).

Recently, due to the enhanced enzymatic activity on proteins and the improved bioactivity of hydrolyzed proteins, ultrasound pretreatment has gained popularity (Qian et al., 2023). Ultrasound alters the secondary and tertiary structures of proteins, leading to partial unfolding and improved enzymatic accessibility (Kaveh, Gholamhosseinpour, et al., 2023; Liu, Iketani, et al., 2022). In a study conducted by Liu, Li, et al. (2022) on hydrolyzed mung bean protein by ultrasound pretreatment, it was found that ultrasonic treatment (546 W, 20 min) reduced the α-helix content and increased the β-sheet content of mung bean protein, and improved the antioxidant properties of hydrolyzed mung bean protein.

The structural stability of peptides must be preserved both during food processing and after consumption. One of the main challenges is the low bioavailability of bioactive peptides due to their susceptibility to gastrointestinal degradation (Cian et al., 2019). Various encapsulation methods have been proposed as a promising strategy to enhance the stability, functional protection, and controlled release of bioactive peptides in food applications (Akbarbaglu et al., 2019; Pateiro et al., 2021). Additionally, encapsulating peptides in nanocarriers offers other benefits, such as improving sensory properties by masking bitterness and increasing solubility (Aguilar-Toalá et al., 2022). The term “nanoencapsulation” refers to the application of encapsulation at the nanometer scale (Bratovcic & Suljagic, 2019).

One encapsulation method is microencapsulation using nanoliposomes. Nanoliposome technology has led to significant advancements in medicine, cosmetics, hygiene, biological membrane research, and even studies on the origin of life. These applications are attributed to several beneficial properties of liposomes, including their ability to mix with both aqueous and non-aqueous media, efficient diffusion, compatibility with fluid environments, optimal size, capacity to encapsulate various materials, and ability to form multilayered structures (Khan et al., 2020).

The primary chemical components of nanoliposomes are phospholipids (Emami et al., 2016). In addition, nanoliposomes may contain other components, such as sterols, within their structure (Eskandari, Sadeghi and Hadi, 2021a, Eskandari, Sadeghi and Hadi, 2021b). The inclusion of sterols in liposomal membranes has three key effects: increasing membrane fluidity, reducing permeability to water-soluble molecules, and stabilizing the membrane in the presence of biological fluids such as plasma (Singh et al., 2019). Cholesterol is the most commonly used sterol in the preparation of lipid vesicles (Perera et al., 2025). However, research indicates that excessive and intermittent cholesterol consumption is linked to cardiovascular diseases and cancer (Xiao et al., 2024).

As an alternative, flaxseed oil can serve as a stabilizer in liposomes. Its fatty acid profile is predominantly alpha-linolenic acid, and it is rich in omega-3, which has been shown to reduce the risk of cardiovascular diseases, high blood pressure, depression, osteoporosis, rheumatoid arthritis, obesity, diabetes, and gastrointestinal disorders (Hoshiar et al., 2021).

Kaveh et al. (2022) reported that by hydrolyzing fenugreek protein with alcalase, pancreatin, pepsin, and trypsin enzymes at hydrolysis time of 30–180 minutes and loading it into liposomal nanovesicles, the antioxidant properties of the hydrolyzed protein and its stability increased after encapsulation. Also, similar results were reported by Khalatbari et al. (2024). They stated that by loading antioxidant peptides from shrimp waste into nanoliposomes with an encapsulation efficiency of 84.67 %, they improved their antioxidant and physicochemical properties.

This research aims to produce hydrolyzed flaxseed meal protein with antioxidant properties using alcalase and pancreatin enzymes in combination with ultrasound technology. Subsequently, the treatment with the highest antioxidant activity was incorporated into a liposomal system using different concentrations of flaxseed oil as a stabilizer. Finally, the physicochemical properties and release characteristics of the formulation were evaluated.

## 2 Materials and methods

### 2.1 Materials

Alcalase, Pancreatin, Trichloroacetic acid, Coomassie brilliant blue (G250), DPPH, Potassium ferricyanide, Ferric chloride, Iron sulfate, Hydrogen peroxide, Iron dichloride, Sodium chloride, Ferrozine, 95 % pure Cholesterol, 97 % Ethanol, Lecithin, Sodium hydroxide, Hydrochloric acid, Potassium dihydrogen phosphate, Phosphoric acid were purchased from the Merck company. The flax seeds (*Linum usitatissimum*) were purchased from a reputable store in Gorgan, Iran.

### 2.2 Preparation of protein concentrate

To remove fat, flaxseed meal powder was mixed with hexane at a 1:4 (*w*/*v*) ratio and stirred for 3 h at room temperature using a shaker set to 150 rpm. The defatted powder was then placed in an oven at 30 °C for 1 h to ensure complete hexane removal. Next, the flaxseed meal powder was mixed with distilled water at a 1:20 ratio, and its pH was adjusted to 10 by adding 1 N NaOH. The mixture was stirred at room temperature for 1 h at 150 rpm. The resulting solution was centrifuged at 4 °C and 8000 rpm for 15 min. The pH of the supernatant was then adjusted to 4 (the isoelectric point of flaxseed meal), followed by another round of centrifugation under the same conditions. Finally, the flaxseed meal protein concentrate was dried using a freeze dryer (Kaveh, Hashemi, et al., 2023).

### 2.3 Ultrasound pretreatment

In the first step, a 5 % flaxseed protein solution was prepared in phosphate buffer (0.2 M, pH 7) and stirred at 25 °C for 24 h. For ultrasound pretreatment, a probe operating at a constant frequency of 200 kHz was applied for 60 s at 40 % intensity. To prevent overheating during the ultrasound process, an ice bath was used (Izanloo & Sadeghi Mahoonak, 2023).

### 2.4 Preparation of flax seed meal protein hydrolysate

Flaxseed meal protein concentrate was dissolved in 0.2 M phosphate buffer at a 5 % (*w*/*v*) concentration. The pH was adjusted to 8 for alcalase and 7.4 for pancreatin. After setting the appropriate temperature for each enzyme (50 °C for alcalase and 37 °C for pancreatin), the enzyme was added according to the RSM design, and the samples were incubated. At specific time intervals (ranging from 30 to 210 min), the enzymes were deactivated by heating at 95 °C for 5 min. The samples were then centrifuged at 8000 rpm for 20 min, and the supernatant was collected and dried using a freeze dryer. It is important to note that enzymatic hydrolysis with alcalase and pancreatin (separately) was performed on both treated and untreated flaxseed meal protein concentrate (Liu, Wang, et al., 2022).

### 2.5 Evaluation of the antioxidant properties

#### 2.5.1 DPPH radical scavenging activity

Briefly, 1.5 mL of the hydrolyzed sample (20 mg/ml) was mixed with 1.5 mL of DPPH ethanol solution (0.15 mM) and vortexed for 30 s. The mixture was then kept in the dark for 45 min before measuring absorbance at 517 nm. Finally, the DPPH radical scavenging activity was calculated using Eq. 1, as described by Akbarbaglu et al. (2025).

(1) $$I\;\left(\%\right)=\left[\frac{\mathrm{Acontrol}-\mathrm{Asample}}{\mathrm{Acontrol}}\text{Ablank}-\text{AsampleAblank}\right]\times 100$$

A _sample_ is the absorbance of the sample and A _control_ is the absorbance of the control.

#### 2.5.2 Total antioxidant capacity

In this method, 0.1 mL of each sample (20 mg/mL) was added to a tube containing 1 mL of total antioxidant reagent, composed of 0.6 M sulfuric acid, 4 mM ammonium molybdate, and 28 mM sodium phosphate. The mixture was then incubated in a 90 °C water bath for 90 min. After incubation, absorbance was measured at 695 nm (Kaveh et al., 2022).

#### 2.5.3 Fe^2+^ chelating activity

1 mL of each sample (20 mg/mL) was mixed with 1.85 mL of distilled water and 0.05 mL of FeCl₂ (2 mM). Next, 0.1 mL of ferrozine solution (5 mM) was added, and the mixture was vortexed for 30 min. The solution was then incubated at 25 °C for 10 min. Finally, absorbance was measured at 562 nm. The chelating activity of the samples was calculated using Eq. 2 (Ambigaipalan et al., 2015).

(2) $${\mathrm{Fe}}^{2+}\;\text{chelating activity}\;\left(\%\right)=\left[\left(A\;\text{control}–A\;\text{sample}/A\;\text{control}\right)\right]\times 100$$

A_control_ is the absorbance of the control and A_sample_ is the absorbance of sample.

### 2.6 Degree of hydrolysis

Briefly, TCA (0.44 M) was mixed with flaxseed meal protein hydrolysate at a 1:1 ratio and incubated at 4 °C for 15 min. The mixture was then centrifuged at 10,000 rpm for 10 min. The protein content in the supernatant, containing 0.22 M TCA, was measured using the Bradford method (1976). Finally, the degree of hydrolysis was calculated using Eq. 3 (Akbarbaglu et al., 2023).

(3) $$\mathrm{DH}\;\left(\%\right)=\text{Protein}\;\left(\mathrm{TCA}+\text{Supernatant}\right)/\text{Protein}\;\left(\text{flaxseed hydrolysate suspension}\right)\times 100$$

### 2.7 Loading of flaxseed meal protein hydrolysate in nanoliposome systems

Liposomes were prepared using the thin-layer hydration method, following the procedure described by Liu et al. (2015). The process involved dissolving 0.09 g of soy lecithin, varying amounts of cholesterol (0.05, 0.04, 0.03, and 0.02 g), 0.02 g of Tween, and varying amounts of flaxseed oil (0.01, 0.02, and 0.03 g) in 10 mL of chloroform (Table 1). A thin film was then formed inside a round-bottom flask using a rotary evaporator at 60 °C and 60 rpm. The flask was kept in a desiccator at room temperature for 12–18 h to ensure complete solvent removal. Next, the dried lipid film was hydrated with 0.2 g of hydrolyzed flaxseed meal protein in 10 mL of distilled water, maintaining a 1:1 ratio between the liposomal wall compound solution and the hydrolyzed protein solution. The mixture was stirred under vacuum at 60 °C for 15 min and then cooled to room temperature. Finally, the solution was subjected to ultrasonic treatment for 10 min using a probe sonicator (100 % power, 0.5 cycle), alternating intervals to prevent temperature rise. The ultrasound process was conducted in cold water to maintain temperature control Table 2.

**Table 1:** Different treatments of nanoliposome loaded with flax seed meal protein hydrolysate.

treatment	Hydrolyzed protein concentration (mg/1 ml)	flaxseed oil (g)	Tween 80 (g)	Cholesterol (g)	lecithin (g)
**L1**	20	0	0.02	0.05	0.09
**L2**	20	0.01	0.02	0.04	0.09
**L3**	20	0.02	0.02	0.03	0.09
**L4**	20	0.03	0.02	0.02	0.09

**Table 2:** Zeta potential, encapsulation efficiency (EE), particle size (DLS) and disintegration index (PDI) of nanoliposomes loaded with hydrolyzed protein of flaxseed meal.

Treatment	DLS (nm)	PDI	Zeta potential (mv)	EE (%)
L1	245.2 ± 54 b	0.57 ± 0.000 a	−7.5 ± 0.75 a	88.9 ± 0.54 c
L2	275.9 ± 78.2 b	0.46 ± 0.007 b	−8.3 ± 0.3 a	95.12 ± 0.29 a
L3	409 ± 63.55 a	0.23 ± 0.007 c	−15.9 ± 0.3 c	95.64 ± 0.23 a
L4	516.3 ± 50.3 a	0.58 ± 0.004 a	−13.8 ± 0.56 b	91.99 ± 0.29 b

#### 2.7.1 Encapsulation efficiency

The microencapsulation efficiency of flaxseed meal protein peptides was determined using a modified version of the method described by Kaveh et al. (2022). Briefly, 1 mL of nanoliposomes was transferred to an Amicon filter with a 10 kDa molecular weight cutoff and centrifuged at 3500 rpm for 10 min. The filtrate was then analyzed to quantify the amount of free (unencapsulated) protein using the Bradford (1976) method. Finally, the encapsulation efficiency was calculated using Eq. 4.

(4) $$\mathrm{EE}\%=\frac{\mathrm{Amount of total protein}-\mathrm{amount of unloaded protein}}{\mathrm{Amount of total protein}}\times 100$$

#### 2.7.2 Poly dispersity index and particle size

The samples were diluted in phosphate-buffered saline (PBS) at a 1:10 ratio. The polydispersity index (PDI) and particle size (*Z*-average) were measured using a dynamic light scattering (DLS) instrument (Wing Sald 2101). Measurements were conducted at 25 °C with a detection angle of 90°.

#### 2.7.3 Zeta potential of liposomes

Briefly, the samples were diluted 1:10 with phosphate buffer (50 mM, pH 7.4). Measurements were conducted at a wavelength of 633 nm, a scattering angle of 90°, and a temperature of 25 °C (Hadian et al., 2013).

#### 2.7.4 Release behavior of nanoliposomes in simulated gastric fluid (SGF) and simulated intestinal fluid (SIF)

Simulated gastric fluid (SGF) containing 0.2 % NaCl and 0.32 % pepsin (pH 2) and simulated intestinal fluid (SIF) containing 50 mM KH₂PO₄ and 0.1 % pancreatin (pH 7.4) were prepared. A 1 mL sample of liposomal suspension was placed in a dialysis bag and incubated in SGF at 37 °C with continuous stirring at 200 rpm for 120 min. Samples (0.5 ml) were collected at 30, 60, 90, and 120 min, with an equal volume of fresh medium added after each collection. The dialysis bag was then transferred to SIF and incubated under the same conditions for 150 min, with sampling at 30, 60, 90, 120, and 150 min (Kaveh et al., 2025). Protein content was analyzed using the Bradford (1976) assay.

#### 2.7.5 Antioxidant activity of loaded liposomes after SGF and SIF

The DPPH radical scavenging activity, total antioxidant capacity, and Fe^2+^ chelating activity of nanoliposome samples were measured at the specified time intervals outlined in Section 2.4 (Akbarbaglu et al., 2025; Ambigaipalan et al., 2015; Kaveh et al., 2022).

#### 2.7.6 FTIR spectroscopy

For FTIR spectroscopy, loaded liposomes were isolated from the liposomal suspension by centrifugation at 18,000 ×*g* for 30 min. The collected liposomes were then freeze-dried. Dried liposomes, hydrolyzed protein, cholesterol, and flaxseed oil were individually mixed with KBr to form tablets. The FTIR spectra of the samples were recorded over a wavelength range of 4000–400 cm^−1^ (Sharma et al., 2015).

#### 2.7.7 Morphological characteristics by scanning electron microscopy (SEM)

The surface morphology of nanoliposomes was analyzed using scanning electron microscopy (SEM) (ZEISS) following the method described by Kaveh et al. (2022).

#### 2.7.8 DSC (differential scanning calorimeter)

Differential scanning calorimetry (DSC) was conducted on liposomal formulations containing hydrolyzed flaxseed meal protein. Thermal analysis was carried out from 10 °C to 600 °C at a scanning rate of 10 °C/minutes under a dry nitrogen atmosphere.

### 2.8 Statistical analysis

Data analysis was conducted using a completely randomized design in SPSS version 16, applying one-way analysis of variance (ANOVA). All tests were performed in triplicate. Duncan's multiple range test was used to compare means and determine statistical significance at *P* < 0.05. Graphs were generated using Excel 2019.

## 3 Results and discussion

### 3.1 Antioxidant activity of hydrolyzed protein

#### 3.1.1 DPPH free radical scavenging activity

**Fig. 1:**
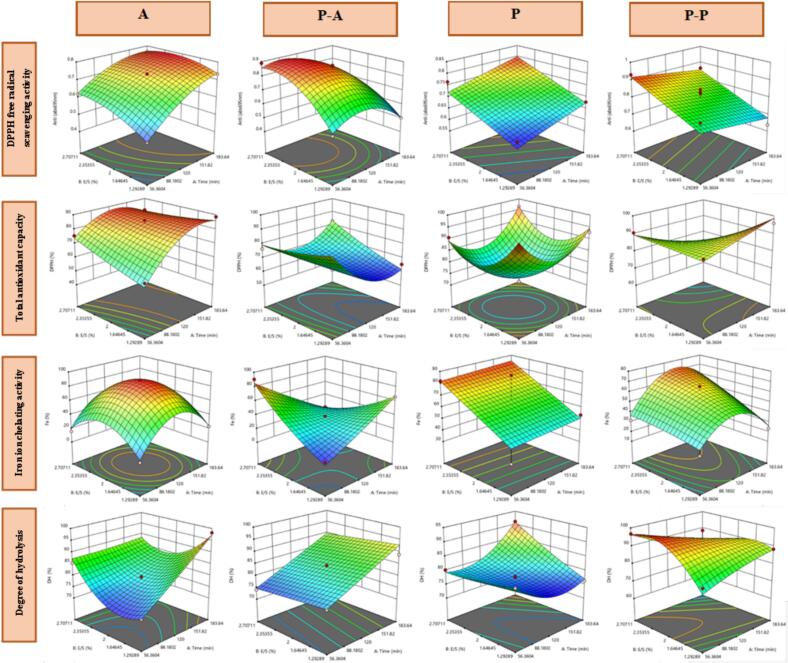
3D diagram of protein hydrolysis analysis of flax seed meal (Treatment A is protein hydrolyzed with alcalase, treatment P-A is protein hydrolyzed with alcalase and ultrasound pretreatment, treatment P is protein hydrolyzed with pancreatin, and treatment P—P is protein hydrolyzed with pancreatin and ultrasound.)

DPPH is a lipid-soluble free radical that absorbs hydrogen from antioxidants, leading to a color change from dark purple to yellow as its concentration decreases (Wang et al., 2017). This makes DPPH scavenging a useful measure of antioxidant activity (Duan et al., 2024). In this study (Fig. 1), enzymatic hydrolysis using pancreatin (Treatment P) and alcalase (Treatment A) without ultrasound pretreatment increased DPPH scavenging as enzyme concentration and hydrolysis time increased, reaching 75.66 % and 74.33 %, respectively. However, excessive hydrolysis may have hindered active amino acids from interacting with DPPH (Alvand et al., 2022; Xue et al., 2008).

With ultrasound-assisted pancreatin hydrolysis (P—P), scavenging efficiency increased with time and decreased pancreatin enzyme-to-substrate ratio, reaching 82.87 %. For ultrasound-alcalase hydrolysis (P-A), the highest DPPH inhibition (79.29 %) occurred in the early stages with low alcalase enzyme concentration, consistent with Kaveh et al. (2018). The improved antioxidant activity in ultrasound-treated pancreatin hydrolysates is likely due to enhanced enzyme access and bioactive peptide release (Fadimu, Gill, Farahnaky and Truong, 2021a, Fadimu, Gill, Farahnaky and Truong, 2021b; Huo et al., 2025). Ultrasound enhances enzymatic hydrolysis by inducing cavitation, which unfolds protein structures, increases enzyme accessibility, and facilitates peptide release (Zhou et al., 2022).Similar results were reported in watermelon seed protein hydrolysis (Wen et al., 2019), beef Liver protein hydrolysis (Duan et al., 2024), chia seed peptides (Madrazo & Campos, 2025) and Muscovy ducks peptides (Gao et al., 2024) where ultrasound pretreatment significantly boosted DPPH scavenging compared to controls. The inhibitory activity of this radical in these studies was 58.89 %, 76.02 %, 86.49 % and, 47.38 % respectively.

#### 3.1.2 Total antioxidant activity

This method relies on the reduction of hexavalent molybdenum to pentavalent molybdenum, which leads to the formation of a green phosphomolybdenum complex in an acidic medium (Farzanfar et al., 2024). The results (Fig. 1) showed that during enzymatic hydrolysis, P and A treatments increased total antioxidant capacity, as indicated by higher absorbance at 695 nm with increasing “time” and “enzyme-to-substrate ratio” (0.660 and 0.648, respectively).

In ultrasound-pretreated samples, the highest antioxidant activity was observed in the early stages of hydrolysis, with increasing enzyme-to-substrate ratio. The absorbance for P—P and P-A treatments was identical at 0.860. This increase is attributed to ultrasound-induced structural unfolding of proteins, which accelerates the release of antioxidant peptides early in hydrolysis (Xue et al., 2017). Additionally, the positive impact of ultrasound pretreatment on P—P and P-A treatments is linked to cavitation, which enhances enzyme accessibility and facilitates peptide release (Qian et al., 2023).

Similar findings have been reported in other studies. Hu and Li (2022) found that ultrasound pretreatment during enzymatic hydrolysis of soy protein increased total antioxidant activity by 72 % compared to the control. Likewise, Pacheco et al. (2025), Wen et al. (2019), Fadimu, Gill, Farahnaky and Truong, 2021a, Fadimu, Gill, Farahnaky and Truong, 2021b, and Muley et al. (2021) observed enhanced antioxidant activity in hydrolyzed pumpkin seed protein, syngonium, lupin protein, and whey protein hydrolysate following ultrasound pretreatment.

#### 3.1.3 Iron ion chelating activity

Fe^2+^ ion acts as a catalyst in the Haber-Weiss reaction, generating hydroxyl radicals that rapidly damage biological molecules. Antioxidants inhibit these radicals by chelating metal ions, including iron (Zudaire et al., 2019). This study (Fig. 1) found that iron ion chelation increased in p-p and A hydrolyzed treatments as the enzyme-to-substrate ratio and hydrolysis time (up to 150 min) increased, reaching 70.39 % and 52.94 %, respectively. In the P treatment, chelation rose to 12.65 % with both variables, while the highest chelation in P-A treatment (13.58 %) occurred early in hydrolysis with a higher enzyme-to-substrate ratio. Extended hydrolysis time negatively affected chelation, possibly due to enzyme inhibition by byproducts (Ovissipour et al., 2009). Ultrasound pretreatment enhanced iron ion chelation by unfolding protein structures and exposing antioxidant-active amino acid residues (Pi et al., 2023). Similar effects were observed in various protein hydrolysates (He et al., 2021; Izanloo & Sadeghi Mahoonak, 2023; Kaveh et al., 2022; Peng et al., 2024). However, Wu et al. (2021) reported no significant impact in abalone viscera protein hydrolysis.

### 3.2 Degree of hydrolysis

The degree of hydrolysis (DH) refers to the percentage of peptide bonds cleaved in a protein during hydrolysis. This process is influenced by factors such as time, temperature, substrate concentration, pH, and enzyme activity (Leni et al., 2020; Vorob'ev, 2025). In this study, the DH of treatments A and P-A hydrolyzed by alcalase enzyme was 75.78 % and 79.06 %, respectively. Similarly, Gerónimo-Alonso et al. (2025) achieved a DH of 81.43 % when hydrolyzing cowpea protein with alcalase. According to the results of this study (Fig. 1), increasing both the alcalase-to-substrate ratio and hydrolysis time led to a higher DH in the P-A treatment. However, in treatment A, which was hydrolyzed with alcalase without pretreatment, these variables had the opposite effect. Research indicates that prolonging hydrolysis time can lead to excessive protein breakdown due to enzyme inactivation, reducing the number of peptide bonds available for hydrolysis and ultimately flattening the hydrolysis curve (Cui et al., 2024). In the P—P treatment, DH increased with a higher pancreatin-to-substrate ratio during the initial minutes of hydrolysis. The DH values for P and P—P treatments were 80.11 % and 80.69 %, respectively. Several studies have shown that ultrasound enhances enzymatic hydrolysis by increasing cell permeability and facilitating enzyme secretion, thereby boosting DH (Ma et al., 2024). Additionally, Cropotova et al. (2024), Olagunju et al. (2018), and Tawalbeh et al. (2023) reported that pancreatin achieves higher hydrolysis rates in fish, pigeon pea, and legume proteins compared to alcalase. This is because pancreatin is a blend of digestive enzymes, including tryptic, chymotryptic, and carboxypeptidase activities, which release polypeptides of varying lengths (Tawalbeh et al., 2023).

### 3.3 Loading of hydrolyzed flaxseed meal protein in nanoliposomes

After comparing the antioxidant activity of different treatments in our study, we found that the protein hydrolyzed with pancreatin enzyme under ultrasound pretreatment for 111.77 min with a 2.26 % ratio of pancreatin enzyme to substrate showed the highest antioxidant activity. As a result, we have chosen this treatment for loading into liposomal carriers.

### 3.4 Investigating the properties of nanoliposomes containing hydrolyzed flaxseed meal protein

#### 3.4.1 Encapsulation efficiency (EE)

The encapsulation efficiency (EE) in this study ranged from 88.90 % to 95.64 % (Table 2), with treatment L3 achieving the highest EE (95.64 %) and treatment L1 the lowest (88.90 %). Increasing flaxseed oil to 0.02 g improved EE; however, further increasing it to 0.03 g while reducing cholesterol to 0.02 g significantly lowered EE (*p* < 0.05). This decline may be attributed to the increased membrane fluidity caused by the unsaturated nature of flaxseed oil, leading to instability and reduced EE (Liang et al., 2007). Similarly, Doiron and Yu (2017) reported that excessive flaxseed oil could disrupt the bilayer structure of liposomes. To prevent this, using a lower amount, such as the 0.02 g in L3, is recommended. Comparable findings were reported by Song et al. (2022) and Yekta et al. (2020) when using flaxseed oil and cholesterol as stabilizers. Additionally, Song et al. (2020), Parhizkary et al. (2024), and Cardoso-Daodu and Ilomuanya (2025) reported EE values of 85.4 %, 84.76 %, and 97 %, respectively, in the microencapsulation of bioactive compounds using nanoliposomes.

#### 3.4.2 Particle size and polydispersity index (PDI)

In this study, the average particle size of liposomes loaded with hydrolyzed flaxseed protein ranged from 245.2 to 516.33 nm (Table 2). Comparing the size of empty liposomes (90.4 nm) to the loaded ones, a noticeable increase in size was observed. Similar findings were reported by Kaveh et al. (2022) and Ganji et al. (2020) in their studies on nanoliposomes containing hydrolyzed fenugreek and hemp proteins. They attributed the size increase to the occupation of liposomal space by protein hydrolysates.

Among the treatments, “Treatment L4” exhibited the largest particle size (516.33 nm), while “Treatment L1” had the smallest (245.2 nm). This trend suggests that increasing the amount of flaxseed oil while reducing cholesterol in the liposomal formulation leads to larger liposome particles. Stabilizing agents such as cholesterol, flaxseed oil, and sterols play a crucial role in modulating the fluidity of phospholipid membranes by preventing the crystallization of phospholipid acyl chains and creating a spatial barrier for their movement (Eskandari et al., 2021). Song et al. (2022) similarly found that higher flaxseed oil concentrations resulted in increased liposomal particle sizes. A similar size increase with higher stabilizer concentrations, including cholesterol, was also reported by Kaveh et al. (2022). The particle size findings in this study were consistent with those reported by Ramezanzade et al. (2017).

The polydispersity index (PDI) is a measure of particle size distribution and uniformity, ranging from 0 to 1 depending on the system type (Romero-Pérez et al., 2010). In this study, the lowest PDI value was observed in Treatment L3 (0.23), indicating a more uniform particle distribution and higher homogeneity compared to other treatments. The PDI decreased as flaxseed oil concentration increased and cholesterol levels decreased, reaching its lowest at Treatment L3. However, in Treatment L4, where cornseed oil concentration increased, the PDI value rose. Excessive flaxseed oil may disrupt the bilayer structure of liposomes (Doiron & Yu, 2017). Song et al. (2022) reported that a PDI below 0.4 indicates a relatively stable liposomal system. The PDI values obtained in this study were comparable to those reported by Yousefi et al. (2023) and Kaveh et al. (2022) in studies on liposomes containing nisin, crocin, and fenugreek.

#### 3.4.3 Zeta potential

In general, a higher absolute zeta potential value indicates stronger repulsion between particles, contributing to the long-term stability of liposomes. Typically, zeta potential values ranging from −30 to +30 mV are considered suitable for colloidal stability (Aguilar-Pérez et al., 2022). In this study, the measured zeta potential values varied between −7.5 and − 15.9 mV (Table 2). The negative values can be attributed to the interaction between hydroxyl and choline groups in cholesterol, which are drawn toward the membrane. This, in turn, attracts the phosphatidyl group to the membrane surface, reducing the zeta potential and leading to the discharge of electrostatic particles (Yekta et al., 2020).

The results indicated that increasing the flaxseed oil content in the liposome formulation up to 0.02 g, while reducing cholesterol to 0.03 g, led to a decrease in zeta potential. However, beyond this point, an increase in zeta potential was observed in the L4 treatment. This pattern aligns with the findings of Song et al. (2022), who demonstrated that adding more flaxseed oil to liposomes resulted in a lower zeta potential. A possible explanation for this trend is the limited capacity of liposomes to encapsulate lipophilic compounds—excessive flaxseed oil may disrupt the liposome bilayer structure (Doiron & Yu, 2017). The zeta potential values obtained in this study were consistent with those reported by Ganji et al. (2020) and Kaveh et al. (2022) for nanoliposomes containing hydrolyzed hemp and fenugreek protein.

#### 3.4.4 Release in the gastric and intestinal conditions

**Fig. 2:**
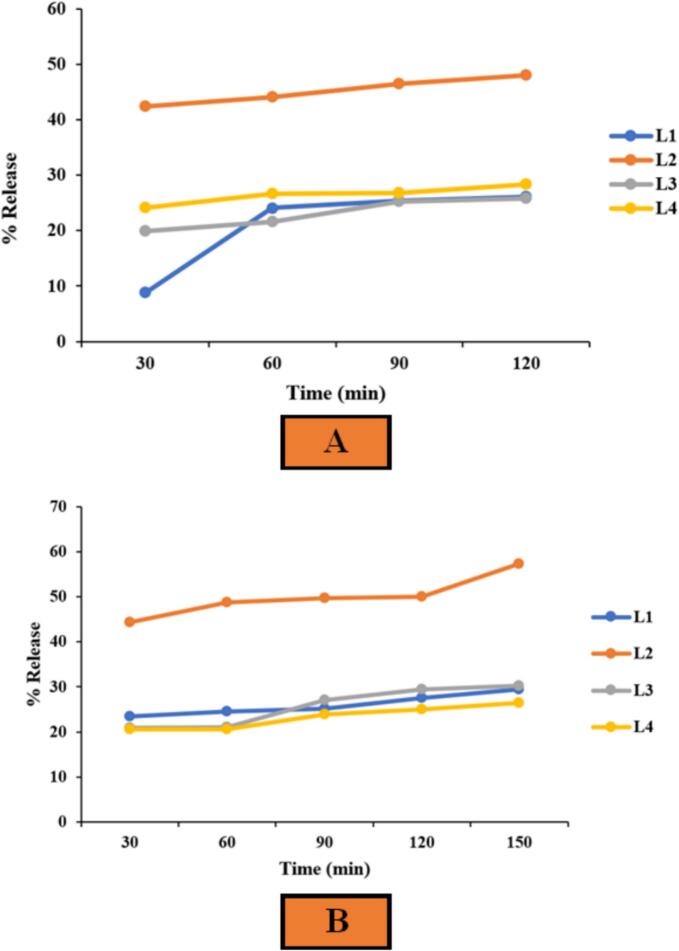
The release rate of different liposome treatments loaded with hydrolyzed flaxseed protein in (A) the simulated gastric condition and (B) simulated intestinal condition.

The release rate of loaded compounds from lipid vesicles depends on various factors, including interactions between the loaded compound and the vesicle membrane, the surfactants used, and the permeability and fluidity of the vesicle membrane (Kaveh et al., 2025). In this study, the release rate of hydrolyzed protein from nanoliposomes was measured under simulated gastric conditions, with the results presented in Fig. 2. The low release rate observed in the gastric environment suggests greater liposome stability and enhanced protection of the bioactive compound, allowing for targeted release. Among the treatments, L1 (without flaxseed oil) exhibited the lowest release rate under simulated gastric conditions, followed by L3 (containing 0.02 g of flaxseed oil and 0.03 g of cholesterol) at 0.92 μg/ml.

Notably, the release rate of treatment L1 showed a steeper increase after 60 min compared to treatment L3. The gradual and controlled release observed in treatment L3 suggests more favorable conditions than other treatments. The release curves of treatments L2, L3, and L4 showed a gentler slope compared to L1, indicating a more controlled release due to the presence of flaxseed oil and cholesterol. Increased membrane fluidity enhances leakage and the release of encapsulated bioactive compounds into the surrounding environment (Kushnazarova et al., 2021). However, an excessive amount of flaxseed oil (0.03 g) may have a destabilizing effect due to its unsaturated structure, leading to increased membrane fluidity and reduced stability (Liang et al., 2007). In order to prevent excessive membrane fluidity and reduced stability, it is better to use smaller amounts of flaxseed oil (0.02 g).

Fig. 2 also presents the release profile under simulated intestinal conditions. Treatment L2 exhibited the highest release (2.5 μg/ml) in the intestinal environment, followed by treatment L3 (1.28 μg/ml). Additionally, treatment L2 showed the highest release rate in the simulated gastric condition among all treatments, highlighting its instability under simulated gastrointestinal conditions.

For all treatments, the concentration of hydrolyzed protein released was higher in the intestinal condition than in the gastric condition. This increased release rate in the intestinal environment is attributed to the presence of pancreatin, which contains multiple enzymes, including lipase, that hydrolyze lipid components in vesicle membranes. Moreover, previous studies have shown that lipid compounds undergo emulsification in the intestinal environment, further enhancing the activity of pancreatin. This leads to membrane degradation and a faster release of encapsulated compounds (Salehi et al., 2022).

In this study, the release rate in the intestinal environment followed a gradual trend, indicating controlled nanoliposome release and highlighting the stabilizing effect of flaxseed oil. The release of hydrolyzed protein from L3-treated nanoliposomes in the simulated digestive environment ranged from 15 % to 35 % over 120 min, a value consistent with the findings of Forutan et al. (2022).

#### 3.4.5 Antioxidant properties of nanoliposomes containing hydrolyzed flaxseed protein

**Fig. 3:**
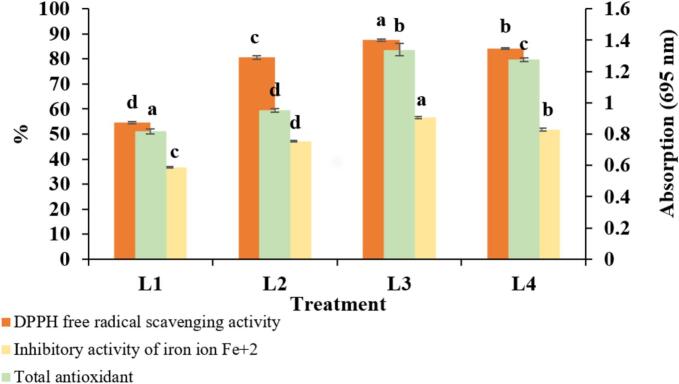
The antioxidant property of different treatments of liposome loaded with hydrolyzed flaxseed protein.

As shown in Fig. 3, increasing the concentration of flaxseed oil led to a rise in DPPH free radical scavenging activity. The highest scavenging activity (87.51 %) was observed in the L3 treatment. The results also revealed a significant difference in DPPH scavenging activity between the L1 treatment and those containing flaxseed oil as a stabilizer in their liposomal walls. This trend suggests that flaxseed oil, with its stabilizing properties, enhanced encapsulation efficiency, thereby increasing DPPH radical inhibition in the treatments containing flaxseed oil (da Rosa Zavareze et al., 2014). Kaveh et al. (2022) also noted that the increased antioxidant capacity of liposomes, with higher cholesterol content, can be attributed to improved encapsulation efficiency. The formation of a complex between anionic radicals and lipophilic DPPH increases the hydrolyzed proteins' ability to trap DPPH radicals.

The highest total antioxidant capacity (1.339 absorbance at 695 nm) was observed in the L3 treatment. In general, increasing the concentration of flaxseed oil as a stabilizer and reducing cholesterol content led to higher total antioxidant activity in treatments L1, L2, and L3. Kaveh et al. (2022) similarly reported an increase in total antioxidant activity with higher cholesterol levels as a stabilizer in the liposomal wall. Modifying the liposome structure by adding stabilizers reduces membrane fluidity and instability, offering better protection for hydrolyzed proteins against adverse environmental conditions. As a result, the antioxidant properties experience fewer changes, and total antioxidant power increases with the higher concentration of stabilizers and improved liposome stability (da Silva Marineli et al., 2014).

Iron ion chelation activity increased across treatments L1, L2, and L3 as flaxseed oil concentration rose. Sarabandi et al. (2019), in their study on nanoliposomes containing flaxseed peptides, and Mosquera et al. (2014), in their work on liposomes with white mouth fish hydrolysates, found that the antioxidant power of bioactive compounds was preserved after encapsulation.

#### 3.4.6 FTIR spectrum

**Fig. 4:**
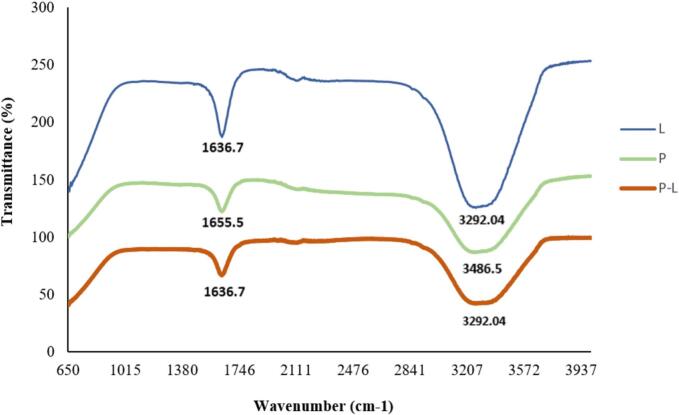
FTIR spectrum of hydrolyzed protein of flaxseed meal (P), empty liposome (L) and loaded liposome with hydrolyzed flaxseed protein (P-L).

The FTIR spectrum of hydrolyzed flaxseed protein, both unloaded and loaded into liposomes (optimal treatment), is presented in Fig. 4. The spectroscopy results indicate that the spectra of empty and loaded liposomes are highly similar, both showing peaks at wavelengths of 1636.7 cm^−1^ and 3292.04 cm^−1^. The peak at 1636.7 cm^−1^ corresponds to amide groups, which include amine (N—H) and ketone (C=O) stretching. Similarly, Hosseini et al. (2017) identified this wavelength as associated with the amide I region (C=O) and random helices. The peak at 3292.04 cm^−1^ is attributed to carboxylic acid stretching and the strong, broad O—H stretching band. Sarabandi et al. (2019) also confirmed the presence of O—H stretching at this wavelength. The broad nature of the O—H stretching band in carboxylic acids is due to their tendency to form hydrogen-bonded dimers. Additionally, carboxylic acid groups contribute to this peak through C—H stretching, while O—H stretching from alcohols and phenols also plays a role.

The FTIR spectrum of hydrolyzed flaxseed protein exhibited peaks at 1000, 1655.5, and 3486.5 cm^−1^. The peak at 1000 cm^−1^ is associated with carbon‑hydrogen (C-H=) bending vibrations of alkenes and aromatic groups. da Rosa Zavareze et al. (2014) and Ferraris et al. (2012) also reported that the initial signals in the spectrum arise from strong OH and NH group vibrations.

In this study, after loading hydrolyzed flaxseed protein into liposomes, peak shifts were observed: from 1655.5 to 1636.7 cm^−1^ and from 3486.5 to 3292.04 cm^−1^. Ferraris et al. (2012) suggested that such shifts to higher or lower frequencies indicate interactions between the encapsulated compound and the liposomal membrane.

Several studies, including those by Ramezanzade et al. (2017), Kaveh et al. (2022), Ganji et al. (2020), Mazloumi et al. (2019), and da Rosa Zavareze et al. (2014), have reported a shift in the N—H stretching peak from 1655.5 to 1636.7 cm^−1^. This shift is attributed to hydrogen bond formation between peptides and phosphatidylcholine, leading to peptide incorporation into the monolayer membrane and the formation of an ionic complex.

#### 3.4.7 Surface structure and FE-SEM scanning electron microscope images

**Fig. 5:**
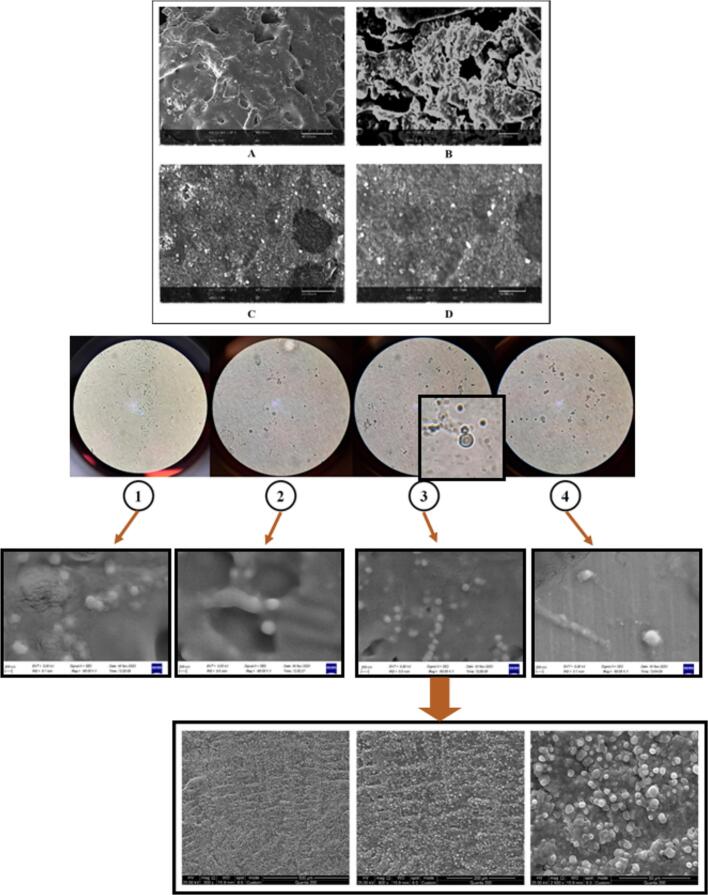
Surface structure images of hydrolyzed protein (A and B correspond to hydrolyzed protein without pretreatment and C and D correspond to hydrolyzed protein with ultrasound pretreatment) and nanoliposomes loaded with hydrolyzed protein of flaxseed (treatments L1 to L4).

The surface structure of the samples was examined using a scanning electron microscope (Fig. 5). In all treatments, nanoliposomes had spherical shapes. Compared to non-spherical particles, spherical particles have more lipid layers and longer distribution paths (Mehnert & Mäder, 2012). Nanoliposomes related to treatment L3 had a smooth, spherical surface and were smaller than other treatments which is in line with the results obtained from DLS analysis. The obtained images were similar to the results of Kaveh et al. (2022) and also Sarabandi et al. (2019) in the loading of fenugreek and flaxseed hydrolyzed proteins in the nanoliposome system, respectively.

#### 3.4.8 DSC

**Fig. 6:**
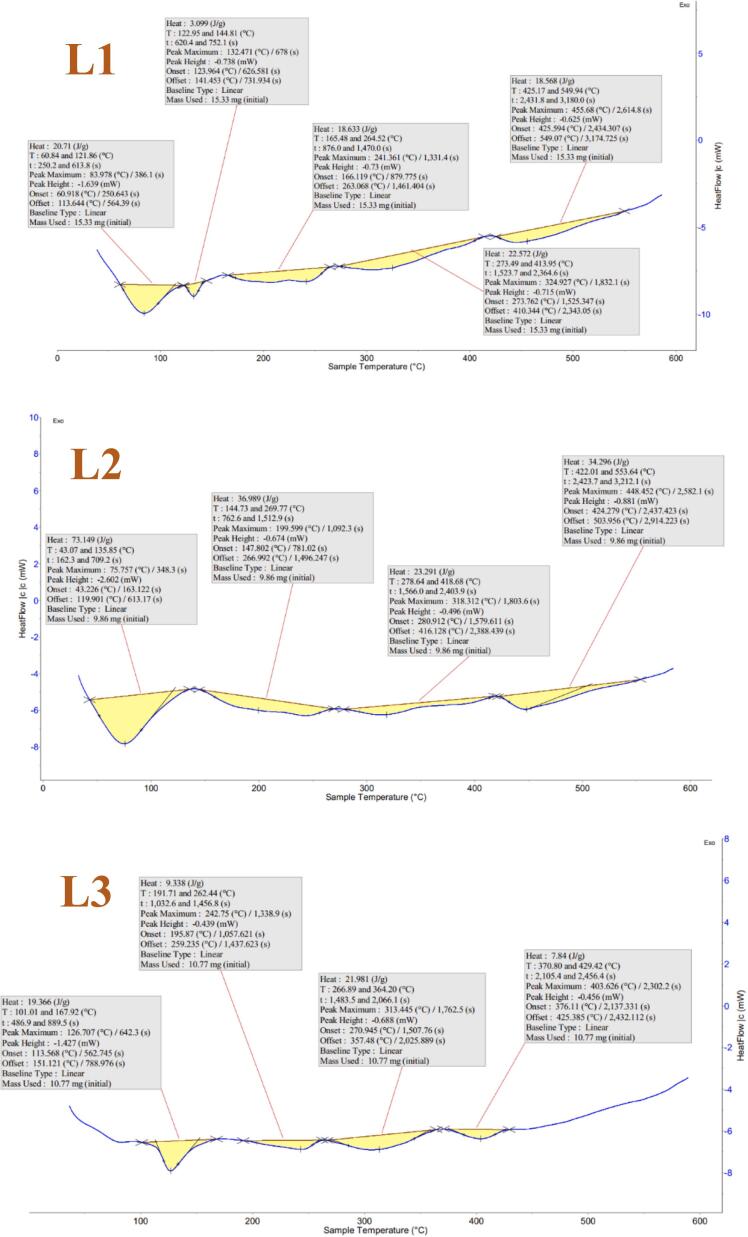

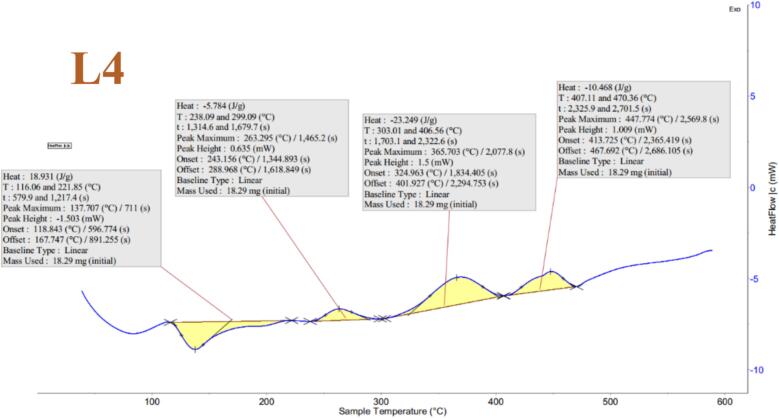
DSC plot of L1, L2, L3 and L4 treatments of liposomes loaded with hydrolyzed flaxseed protein.

The impact of temperature on the phase changes of liposomal samples L1, L2, L3, and L4 was examined by heating them in the temperature range of 0–600 °C (see Fig. 6). The results revealed that crystallization phase change was observed in the sample without flaxseed oil at a temperature of 83.9 °C. However, as the amount of flaxseed oil in the formulation of samples L2, L3, and L4 gradually increased, the peak of crystallization phase change was observed at temperatures of 75.7, 126.7, and 137.7 °C, respectively. This increase in thermal stability can be attributed to the interaction of flaxseed oil with lecithin phospholipids, resulting in the formation of a compact and rigid structure, reducing molecular chain mobility (Lee et al., 2017). Additionally, in sample L4, a melting phase change peak was observed at 365 °C in the graph, indicating the instability of L4 compared to other samples. The excessive increase of oil in the phospholipid bilayers may have disrupted their ordered structure, reducing membrane stiffness and Tm (Ghaleshahi & Rajabzadeh, 2020). Furthermore, in samples L1 and L2, the peaks were wider than in sample L3, suggesting greater thermal stability in L3.

## 4 Conclusion

This study found that hydrolyzed flaxseed meal protein exhibits strong antioxidant activity, including iron ion chelation, DPPH free radical scavenging, and overall antioxidant capacity. Key factors influencing its antioxidant potential included the **enzyme-to-substrate ratio, enzyme type**, and **hydrolysis duration**. Additionally, **ultrasound pretreatment** significantly enhanced the antioxidant power of the hydrolyzed protein.

Among the tested conditions, the combination of **pancreatin enzyme** and **ultrasound pretreatment** (with a hydrolysis time of 111.77 min and an enzyme-to-substrate ratio of 2.26 %) resulted in the highest antioxidant activity. Furthermore, replacing cholesterol with **0.02 g of flaxseed oil** in the nanoliposome formulation improved its nutritional profile while ensuring stability under simulated digestive conditions. This formulation also helped preserve the antioxidant properties of hydrolyzed flaxseed meal protein and facilitated its controlled release.

FTIR analysis confirmed the **successful encapsulation of hydrolyzed protein in nanoliposomes**, achieving a high microencapsulation efficiency of **95.64 %**. Given its strong antioxidant potential, and pending further clinical studies, nanoliposomes containing hydrolyzed flaxseed meal protein could be effectively used as plant-based antioxidants in food formulations to develop functional and health-promoting products.

## CRediT authorship contribution statement

**Faezeh Farzanfar:** Writing – original draft, Software, Investigation, Formal analysis, Data curation. **Alireza Sadeghi Mahoonak:** Writing – review & editing, Validation, Supervision, Methodology, Conceptualization. **Mohammad Ghorbani:** Validation, Methodology. **Seyed Hossein Hosseini Ghaboos:** Validation. **Shima Kaveh:** Writing – review & editing, Software, Resources, Methodology.

## Declaration of competing interest

The authors declare that they have no known competing financial interests or personal relationships that could have appeared to influence the work reported in this paper.

## Data Availability

Data will be made available on request.
